# Supplementary material for: Ventral pallidum neurons dynamically signal relative threat
Source: Commun Biol. 2021 Jan 8;4:43. doi: 10.1038/s42003-020-01554-4 (PMC7794503; doi:10.1038/s42003-020-01554-4)
Supplement: Supplementary file 2 — Supplementary Information [file 42003_2020_1554_MOESM2_ESM.pdf]

Title

Ventral pallidum neurons dynamically signal relative threat

Mahsa Moaddab*, Madelyn H. Ray and Michael A. McDannald*

*Corresponding Authors

Boston College
Department of Psychology & Neuroscience
140 Commonwealth Avenue
514 McGuinn Hall
Chestnut Hill, MA, USA

Please address correspondence to M.M. (moaddab@bc.edu) or M.A.M. (michael.mcdannald@bc.edu).

Acknowledgements

Research reported in this publication was supported by the National Institute of Mental Health of the National Institutes of Health under Award Numbers MH113053 and MH117791. The content is solely the responsibility of the authors and does not necessarily represent the official views of the National Institutes of Health. We thank Bret Judson and the Boston College Imaging Core for infrastructure and support.

Author contributions

M.H.R. bred the rats, M.M. and M.A.M. designed the experiment, M.M. performed the surgeries and M.M. collected the single-unit and behavioral data. M.M. and M.A.M. interpreted/analyzed the data and wrote the manuscript, M.M., M.A.M. and M.H.R edited and approved the final manuscript.

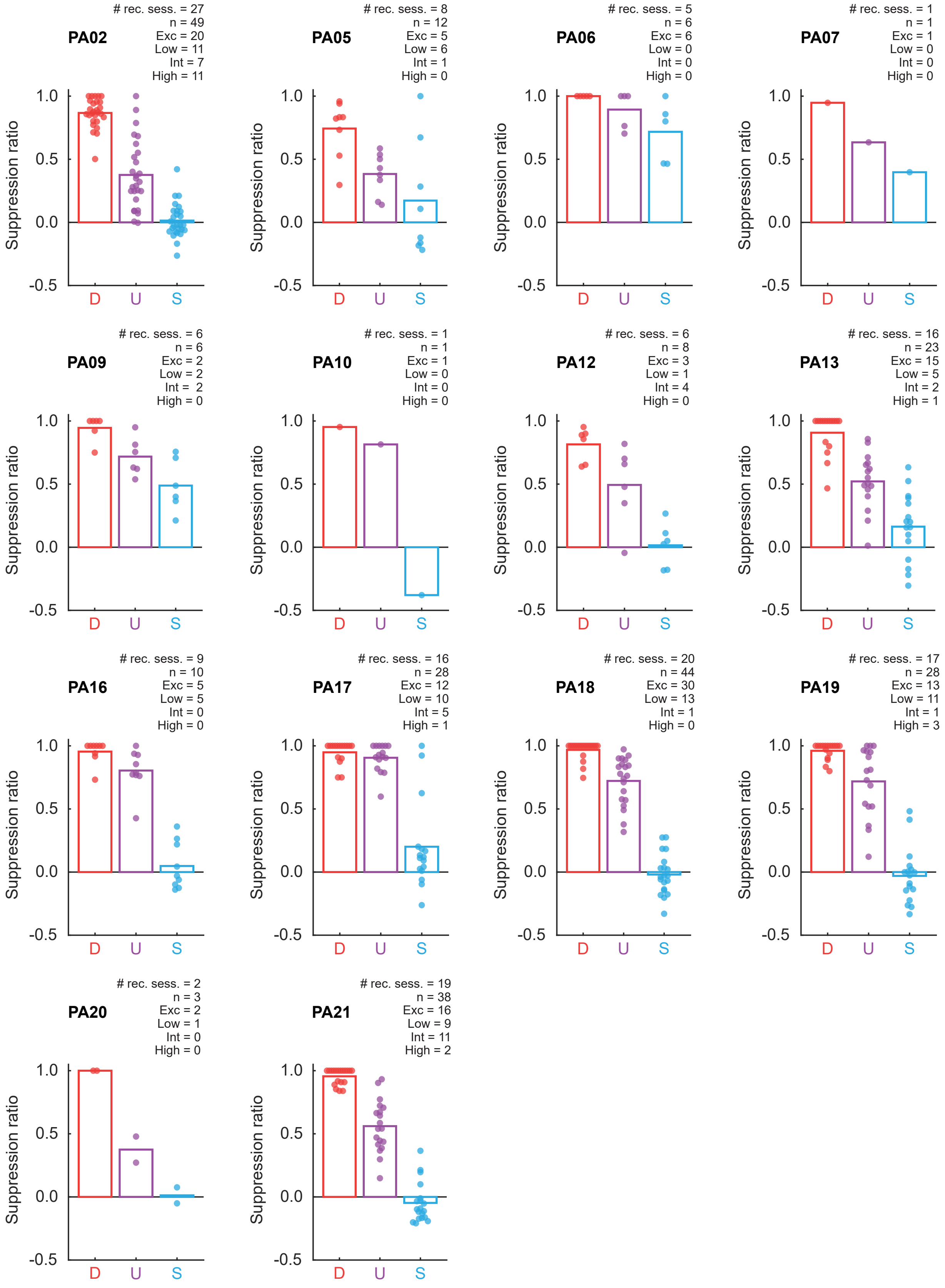

Supplementary Figure 1. Individual fear discrimination and recording summary. Mean (bar) and individual session (data points) suppression ratio for each cue (D, danger, red; U, uncertainty, purple; S, safety, blue) is shown for each individual for all recording sessions with cue-responsive neurons. Animal identity is shown in the top left. For each individual, the number of recording sessions with cue-responsive neurons, the number of cue-responsive neurons, and the number of neurons in each population (Exc, cue-excited; Low, Low firing; Int, Intermediate firing; High, High firing) are provided.

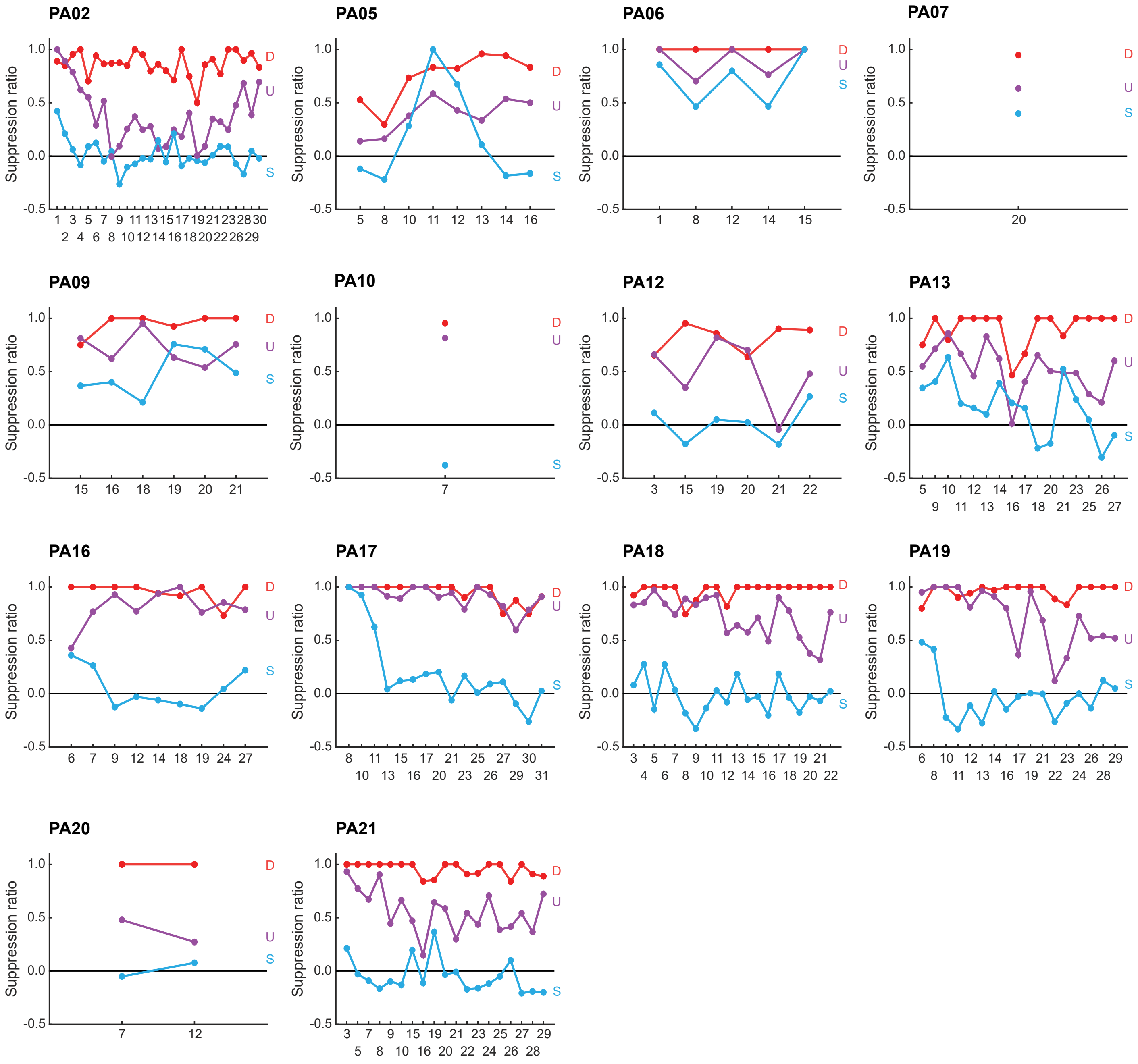

Supplementary Figure 2. Individual fear discrimination session by session. Suppression ratios for each cue (D, danger, red; U, uncertainty, purple; S, safety, blue) are shown for each individual for all recording sessions with cue-responsive neurons. Animal identity is shown in the top left. Recording session number on the x-axis.

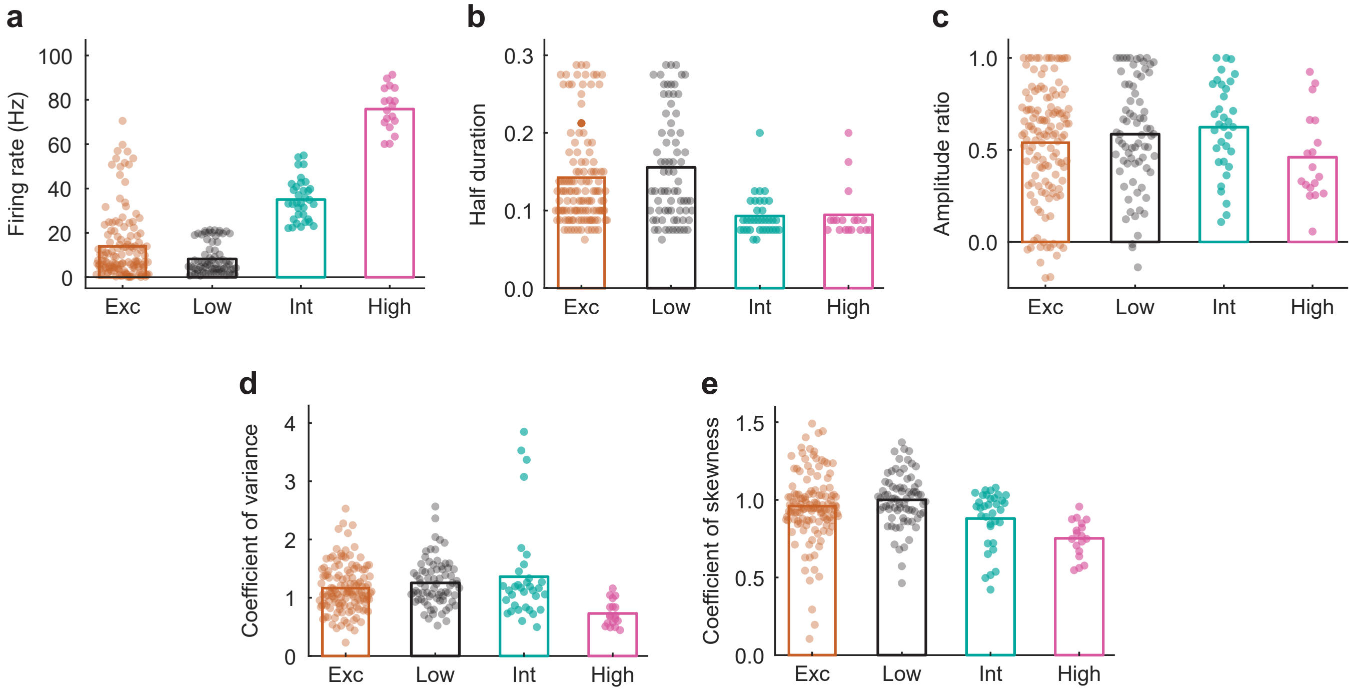

Supplementary Figure 3. Firing and waveform characteristics. Mean (bar) and individual (data points) (**a**) firing rate (**b**) waveform half duration (**c**) waveform amplitude ratio (**d**) coefficient of variance, and (**e**) coefficient of skewness during a 10 s baseline period just prior to cue onset for cue-excited (Exc, n = 131, maroon), Low firing (Low, n = 74, black), Intermediate firing (Int, n = 34, turquoise), and High firing (High, n = 18, pink) neurons.

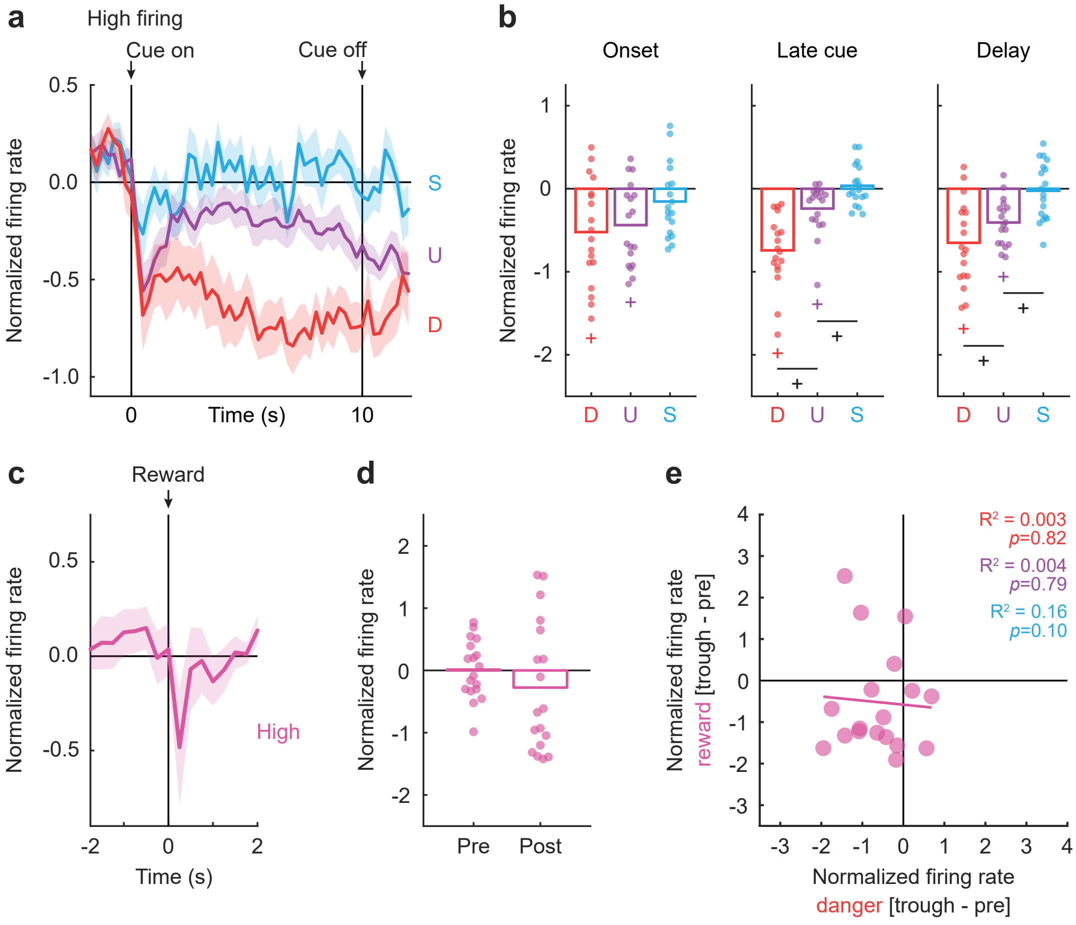

Supplementary Figure 4. High firing neurons show differential cue firing during the late cue and delay periods. (**a**) Mean ± SEM normalized firing rate to danger (D, red), uncertainty (U, purple) and safety (S, blue) is shown from 2 s prior to cue onset to 2 s following cue offset for the High firing neurons (n = 18). Cue onset and offset are indicated by vertical black lines. SEM is indicated by shading. (**b**) Mean (bar) and individual (data points), normalized firing rate during the first 1 s cue interval (onset, left), the last 5 s cue interval (late cue, middle), and 2 s following cue offset (delay, right) are shown for each cue (D, danger, red; U, uncertainty, purple; and S, safety, blue). +95% bootstrap confidence interval for differential cue firing rate does not contain zero. +95% bootstrap confidence interval for normalized firing rate does not contain zero (colored plus signs). (**c**) Mean ± SEM normalized firing rate to reward is shown 2 s prior to 2 s following reward (advancement of pellet dispenser). SEM is indicated by shading. (**d**) Mean (bar) and individual (data points), normalized firing rate are shown during 500 ms interval prior (pre) to and 500 ms interval after (post) the reward delivery for High firing neurons (pink). (**e**) Mean normalized firing rate to reward (250 ms prior to reward delivery to 250 ms following reward delivery, [trough - pre]) vs. danger (the second 250 ms of cue, [trough - pre], red) is plotted. Trendline, the square of the Pearson correlation coefficient (R^2) and associated p value (*p*) are shown. R^2 and *p* for uncertainty (purple) and safety (blue) are provided.

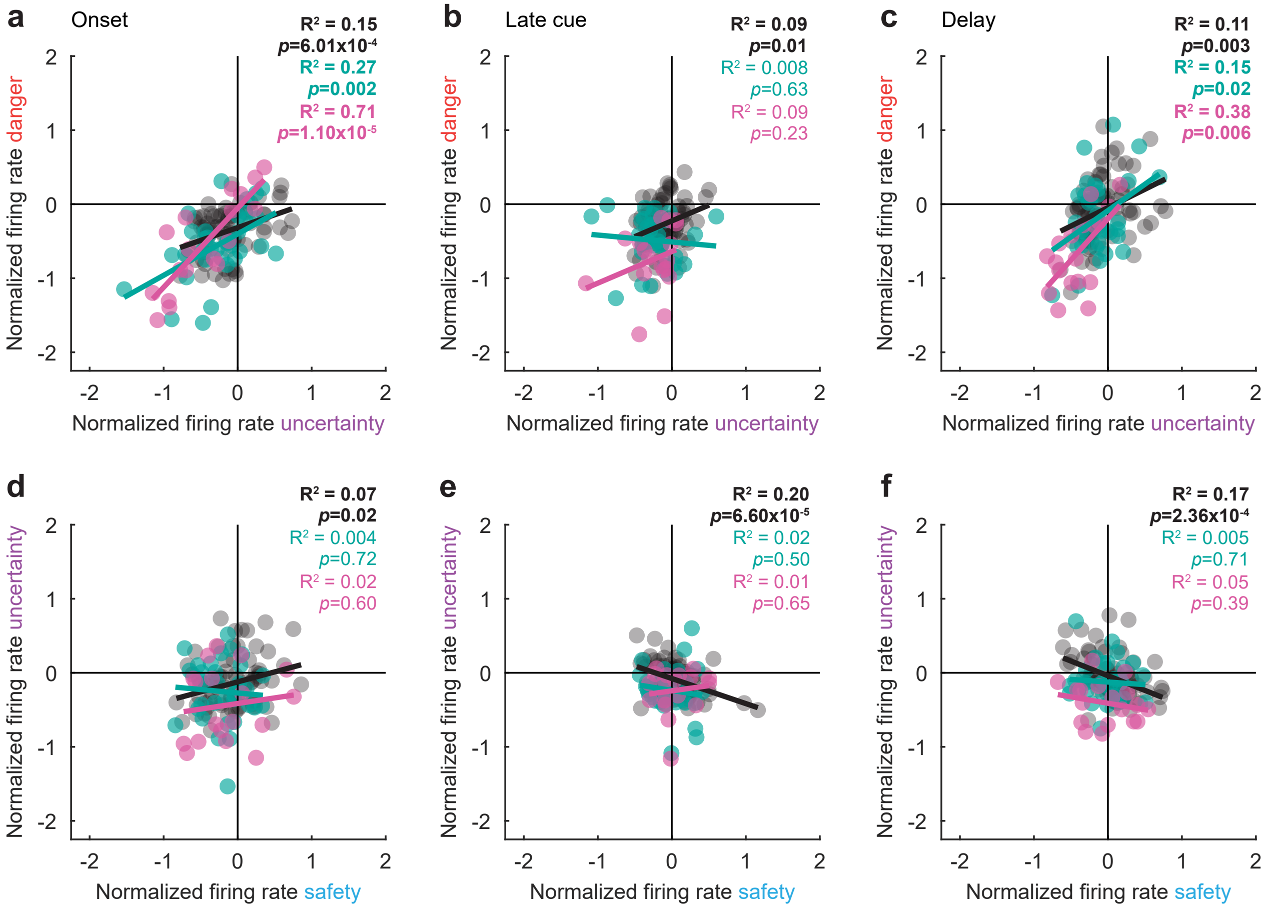

Supplementary Figure 5. Cue firing relationships. Scatterplots for mean normalized firing rate to danger (red) vs. uncertainty (purple) are shown for (**a**, onset) the first 1 s cue interval, (**b**, late cue) the last 5 s cue interval, and (**c**, delay) 2 s following cue offset for the cue-inhibited neurons (Low firing, n = 74, black; Intermediate firing, n = 34, turquoise; High firing, n = 18, pink). Trendline, the square of the Pearson correlation coefficient (R^2) and associated p value (*p*) are shown for each cluster. (**d-f**) Scatterplots for mean normalized firing rate to uncertainty (purple) vs. safety (blue) shown, as in a-c.

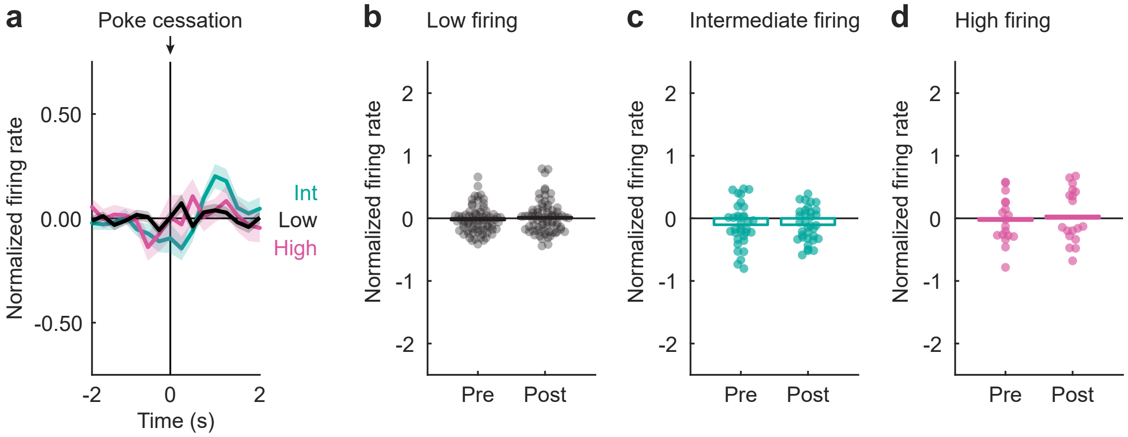

Supplementary Figure 6. Nose poke cessation is insufficient to drive activity of Low, Intermediate and High firing neurons. (**a**) Mean ± SEM normalized firing rate is shown 2 s prior to and 2 s after nose poke cessation for the Low (Low, n = 74, black), Intermediate (Int, n = 34, turquoise), and High (High, n = 18, pink) firing neurons. SEM is indicated by shading. All cue-inhibited neurons were unresponsive to nose poke cessation. Nose poke cessation is indicated by black arrow. (**b-d**) Mean (bar) and individual (data points), normalized firing rate for (**b**) Low, (**c**) Intermediate, and (**d**) High firing neurons are shown during 500 ms interval prior (pre) to and 500 ms interval after (post) nose poke cessation. Colors maintained from a.

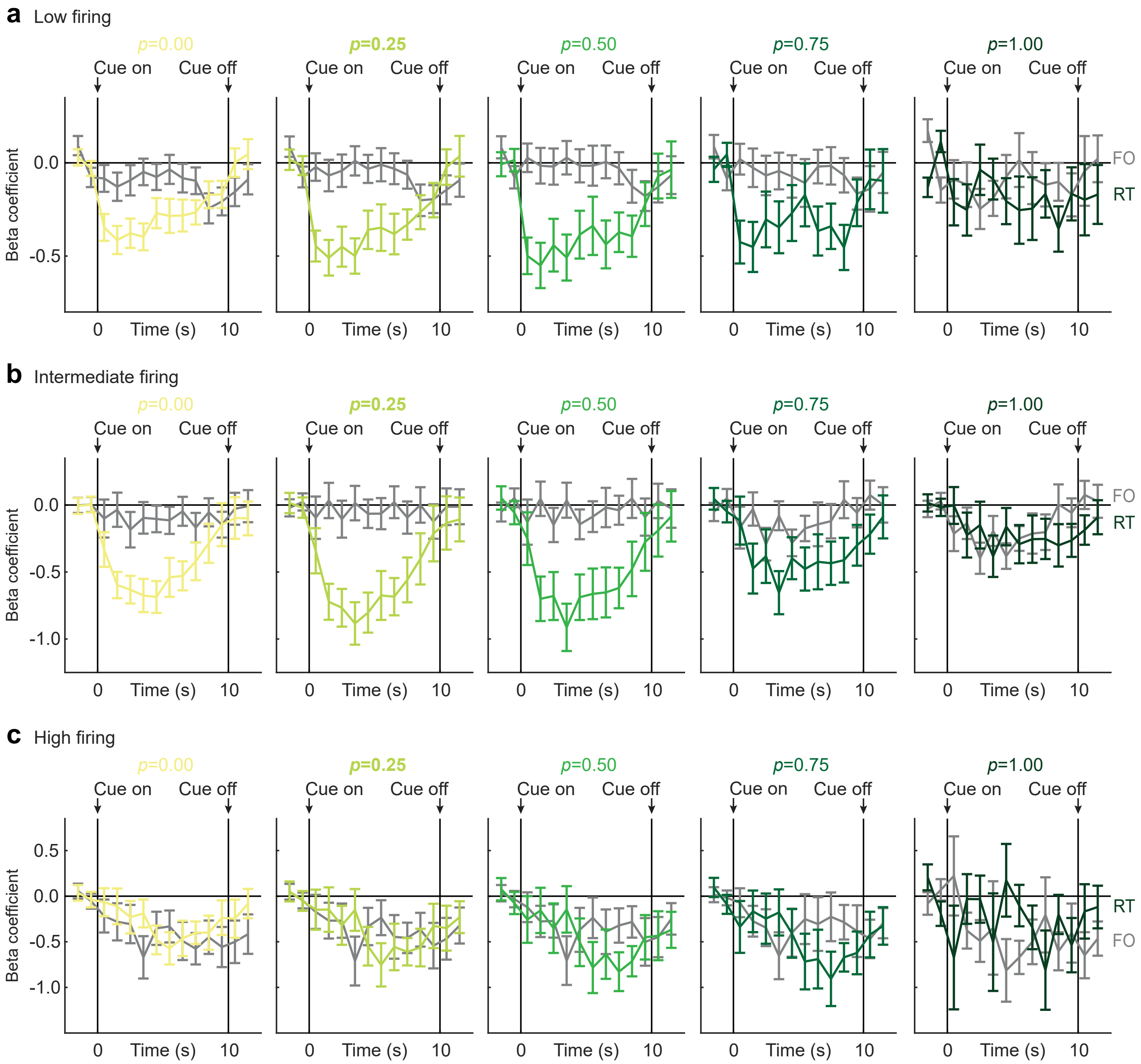

Supplementary Figure 7. Complete regression outcomes for Low, Intermediate and High firing neurons. (**a**) Mean ± SEM beta coefficients are shown for each regressor (RT, relative threat; FO, fear output), from 2 s prior to cue onset to 2 s following cue offset in 1 s bins, for each uncertainty assignment from 0 to 1 in 0.25 increments (0.00, 0.25, 0.50, 0.75, and 1.00), for the Low firing neurons (n = 74). Cue onset and offset are indicated by vertical black lines. (**b** and **c**) Mean ± SEM beta coefficients for (**b**) Intermediate (n = 34) and (**c**) High (n = 18) firing neurons, shown as in a. Low and Intermediate firing neurons more strongly signal relative threat, compared to fear output, across all assignments. High firing neurons signal a mixture of relative threat and fear output.

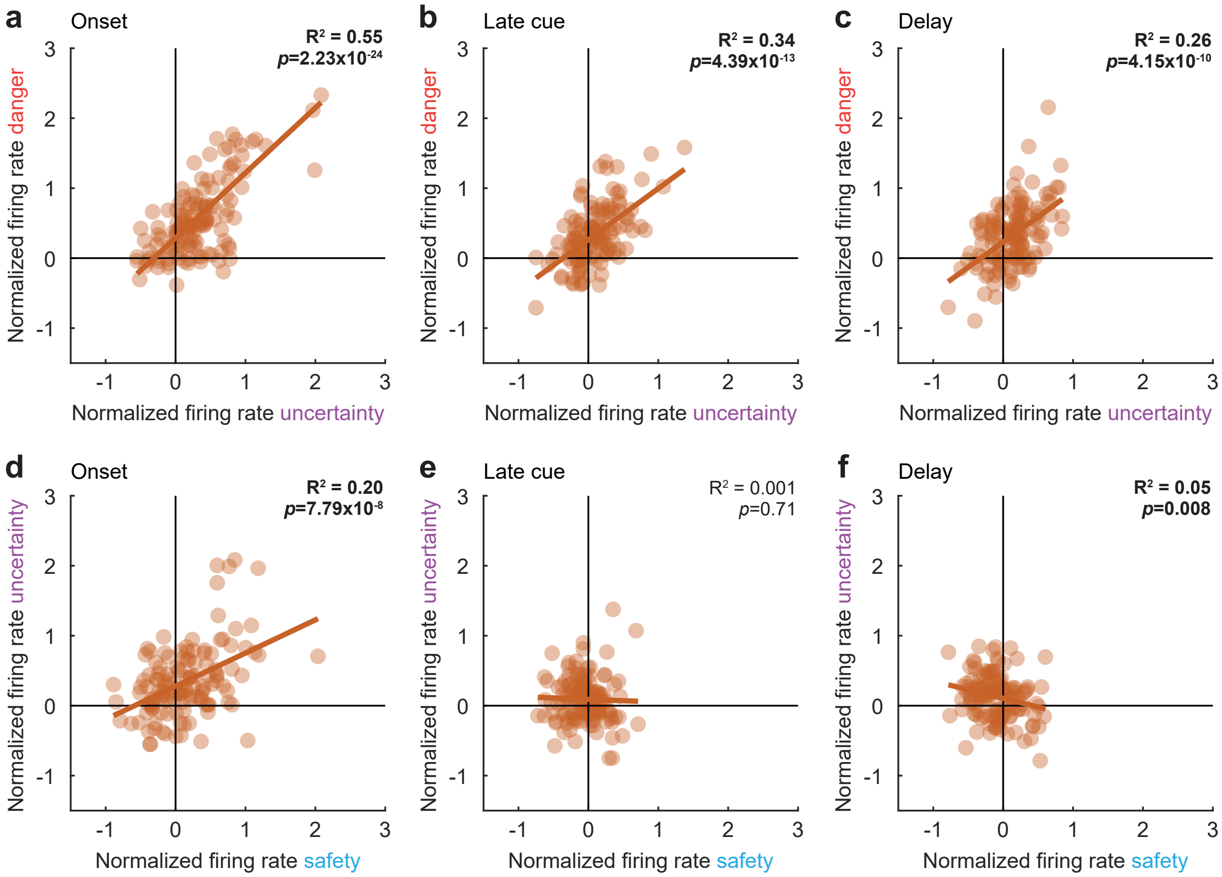

Supplementary Figure 8. Cue firing relationships in cue-excited neurons. Scatterplots for mean normalized firing rate to danger (red) vs. uncertainty (purple) are shown for (**a,** onset) the first 1 s cue interval, (**b,** late cue) last 5 s cue interval, and (**c,** delay) 2 s following cue offset for the cue-excited neurons (n = 131, maroon). Trendline, the square of the Pearson correlation coefficient (R^2) and associated p value (*p*) are shown. (**d**-**f**) Scatterplots for mean normalized firing rate to uncertainty (purple) vs. safety (blue) shown, as in a-c.

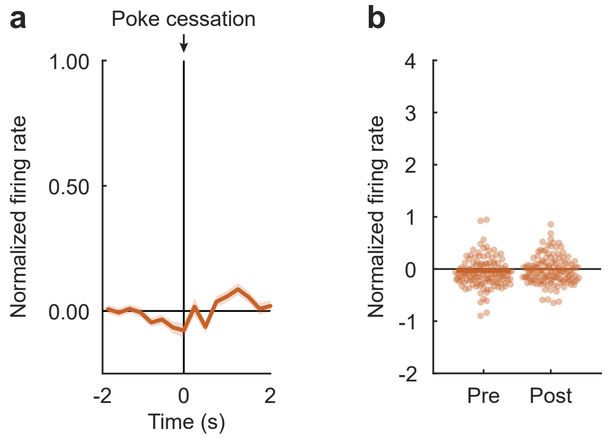

Supplementary Figure 9. Nose poke cessation is insufficient to drive activity of cue-excited neurons. (**a**) Mean ± SEM normalized firing rate is shown 2 s prior to and 2 s after nose poke cessation for cue-excited neurons (n = 131, maroon). Nose poke cessation is indicated by black arrow. SEM is indicated by shading. (**b**) Mean (bar) and individual (data points), normalized firing rate for cue-excited neurons are shown during 500 ms interval prior (pre) to and 500 ms interval after (post) nose poke cessation. Color maintained from a.

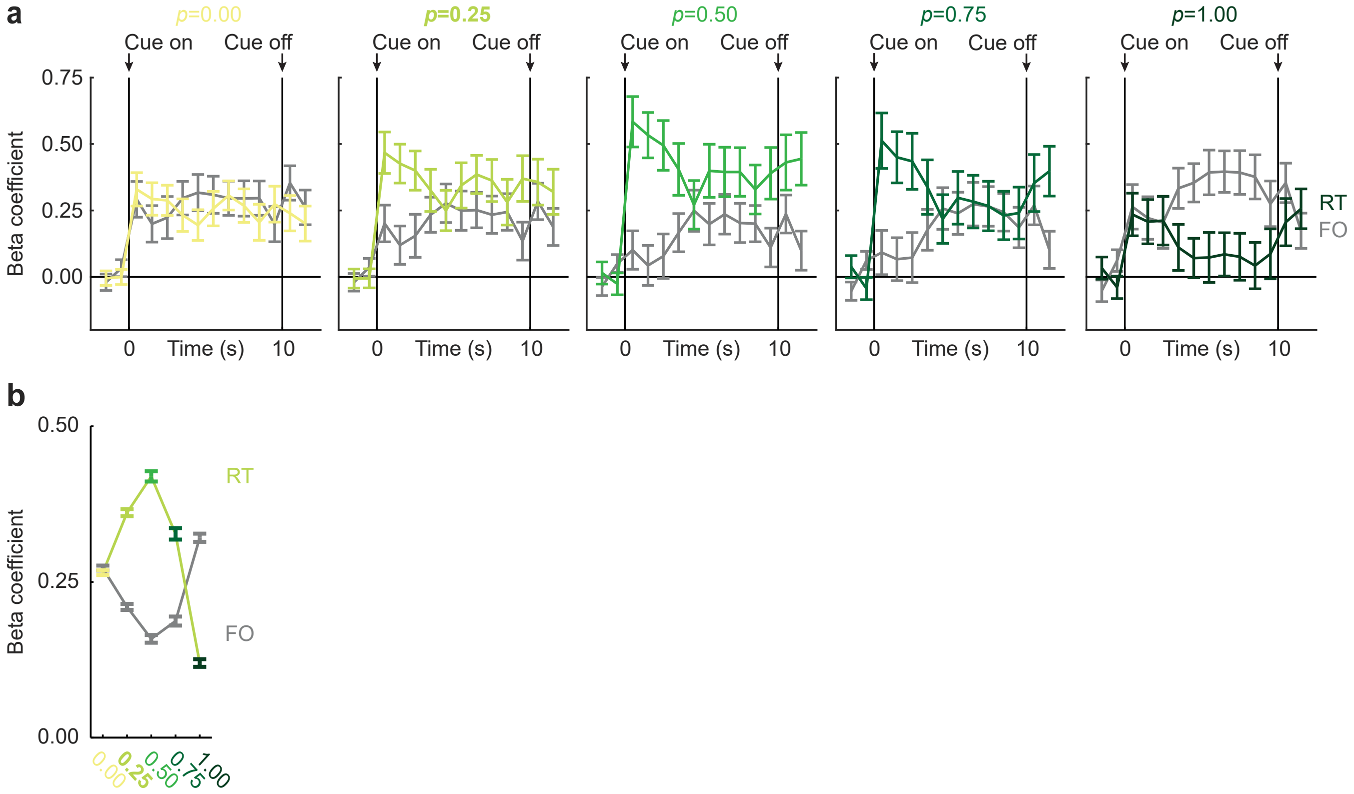

Supplementary Figure 10. Cue-excited neurons signal relative threat and fear output. Mean ± SEM beta coefficients are shown for each regressor (RT, relative threat; FO, fear output), from 2 s prior to cue onset to 2 s following cue offset in 1 s bins, for each uncertainty assignment from 0 to 1 in 0.25 increments (0.00, 0.25, 0.50, 0.75, and 1.00), for the cue-excited neurons (n = 131). Cue onset and offset are indicated by vertical black lines. (**b**) Mean ± SEM beta coefficients are shown for each regressor (RT, relative threat; FO, fear output), during the 10 s cue, for each assignment from 0 to 1 in 0.25 increments, for the cue-excited neurons. Cue-excited neurons signal a mixture of relative threat and fear output across all assignments.
