# Supplementary material for: Ventral pallidum neurons dynamically signal relative threat
Source: Commun Biol. 2021 Jan 8;4:43. doi: 10.1038/s42003-020-01554-4 (PMC7794503; doi:10.1038/s42003-020-01554-4)
Supplement: Supplementary file 3 — Reporting Summary [file 42003_2020_1554_MOESM3_ESM.pdf]

nature research

Corresponding author(s): Michael A. McDannald and Mahsa Moaddab

Last updated by author(s): 11/19/2020

Reporting Summary

Nature Research wishes to improve the reproducibility of the work that we publish. This form provides structure for consistency and transparency in reporting. For further information on Nature Research policies, see our Editorial Policies and the Editorial Policy Checklist.

Statistics

For all statistical analyses, confirm that the following items are present in the figure legend, table legend, main text, or Methods section.

n/a	Confirmed	
☐	☒	The exact sample size (n) for each experimental group/condition, given as a discrete number and unit of measurement
☐	☒	A statement on whether measurements were taken from distinct samples or whether the same sample was measured repeatedly
☐	☒	The statistical test(s) used AND whether they are one- or two-sided<br>*Only common tests should be described solely by name; describe more complex techniques in the Methods section.*
☐	☒	A description of all covariates tested
☐	☒	A description of any assumptions or corrections, such as tests of normality and adjustment for multiple comparisons
☐	☒	A full description of the statistical parameters including central tendency (e.g. means) or other basic estimates (e.g. regression coefficient) AND variation (e.g. standard deviation) or associated estimates of uncertainty (e.g. confidence intervals)
☐	☒	For null hypothesis testing, the test statistic (e.g. F, t, r) with confidence intervals, effect sizes, degrees of freedom and P value noted<br>*Give P values as exact values whenever suitable.*
☒	☐	For Bayesian analysis, information on the choice of priors and Markov chain Monte Carlo settings
☒	☐	For hierarchical and complex designs, identification of the appropriate level for tests and full reporting of outcomes
☒	☐	Estimates of effect sizes (e.g. Cohen's d, Pearson's r), indicating how they were calculated

Our web collection on statistics for biologists contains articles on many of the points above.

Software and code

Policy information about availability of computer code

Data collection	Data were acquired with software from Med Associates and Plexon.
Data analysis	Data were analyzed with Matlab 2018b.

For manuscripts utilizing custom algorithms or software that are central to the research but not yet described in published literature, software must be made available to editors and reviewers. We strongly encourage code deposition in a community repository (e.g. GitHub). See the Nature Research guidelines for submitting code & software for further information.

Data

Policy information about availability of data

All manuscripts must include a data availability statement. This statement should provide the following information, where applicable:

- Accession codes, unique identifiers, or web links for publicly available datasets
- A list of figures that have associated raw data
- A description of any restrictions on data availability

Full electrophysiology data set and basic Matlab code will be uploaded to http://crcns.org/ upon acceptance for publication.

Field-specific reporting

Please select the one below that is the best fit for your research. If you are not sure, read the appropriate sections before making your selection.

☒ Life sciences ☐ Behavioural & social sciences ☐ Ecological, evolutionary & environmental sciences

For a reference copy of the document with all sections, see nature.com/documents/nr-reporting-summary-flat.pdf

Life sciences study design

All studies must disclose on these points even when the disclosure is negative.

Sample size	We set out to collect single units from >10 individuals to ensure single-unit types observed were representative of the ventral pallidum.
Data exclusions	Single units that were not held for the duration of the session were excluded from analysis. No sessions were excluded on the basis of behavior or neural activity.
Replication	Sufficient information is provided to replicate probe targeting of the ventral pallidum and fear discrimination.
Randomization	Auditory identity of cues was counterbalanced between rats.
Blinding	Experiment design was within-subjects, so all subjects experienced all conditions. Behavioral data were not hand scored.

Reporting for specific materials, systems and methods

We require information from authors about some types of materials, experimental systems and methods used in many studies. Here, indicate whether each material, system or method listed is relevant to your study. If you are not sure if a list item applies to your research, read the appropriate section before selecting a response.

Materials & experimental systems

n/a	Involved in the study	
☐	☒	Antibodies
☒	☐	Eukaryotic cell lines
☒	☐	Palaeontology and archaeology
☐	☒	Animals and other organisms
☒	☐	Human research participants
☒	☐	Clinical data
☒	☐	Dual use research of concern

Methods

n/a	Involved in the study	
☒	☐	ChIP-seq
☒	☐	Flow cytometry
☒	☐	MRI-based neuroimaging

Antibodies

Antibodies used	Primary antibody: Rabbit anti-substance P, Immunostar, Cat # 20064, Lot # # 2013001; Secondary antibody: Alexa Fluor 594 donkey anti-rabbit, Jackson ImmunoResearch Laboratories, Code # 711-585-152
Validation	https://www.citeab.com/antibodies/677126-20064-substance-p-antibody?utm_campaign=Widget+All +Citations&utm_medium=Widget&utm_source=ImmunoStar&utm_term=ImmunoStar

Animals and other organisms

Policy information about studies involving animals; ARRIVE guidelines recommended for reporting animal research

Laboratory animals	Rat, Long Evans, Female
Wild animals	NA
Field-collected samples	NA
Ethics oversight	All protocols were approved by the Boston College Animal Care and Use Committee and all experiments were carried out in accordance with the NIH guidelines regarding the care and use of rats for experimental procedures.

Note that full information on the approval of the study protocol must also be provided in the manuscript.
